# The CoGenT3 Study: Examining Gender’s Impact on Education and Cognition Trends in Three American Generations

**DOI:** 10.1093/geroni/igaa057.2441

**Published:** 2020-12-16

**Authors:** Shana Stites, Hannah Cao, Jeanine Gill, Kristin Harkins, Jonathan Rubright, Jason Flatt

**Affiliations:** 1 University of Pennsylvania, Philadelphia, Pennsylvania, United States; 2 National Board of Medical Examiners, Philadelphia, South Carolina, United States; 3 University of Nevada, Las Vegas, School of Public Health, Las Vegas, California, United States

## Abstract

How older adults protect their cognitive health, reduce their risk for cognitive decline, and manage cognitive changes vary for men and women. To advance what is known about these differences and to promote inclusion of sexual and gender minorities in research, we are developing an empirically-informed research framework for studying gender effects in aging and Alzheimer’s research. In this presentation, we describe the framework informing our approach and present results from analyses of gender effects in The Health and Retirement Study that examine gender differences in the associations observed between education and cognitive measures in older adults. Our findings show gender’s effects on education vary in direction and magnitude as gender norms changed over time. Although college education serves as a factor protective against cognitive decline, characteristics of who achieves a four-year college degree change over time. We discuss the implications of our results for aging and Alzheimer’s disease research.

